# Combined benefits of fermented washed rice water and NPK mineral fertilizer on plant growth and soil fertility over three field planting cycles

**DOI:** 10.1016/j.heliyon.2023.e20213

**Published:** 2023-09-15

**Authors:** Abba Nabayi, Christopher Boon Sung Teh, Ali Kee Zuan Tan, Ngai Paing Tan, Dóra Beke

**Affiliations:** a Department of Land Management, Faculty of Agriculture, Universiti Putra Malaysia, 43400 UPM, Serdang, Selangor, Malaysia; b Department of Soil Science, Faculty of Agriculture, Federal University Dutse, Nigeria. PMB 7156, Ibrahim Aliyu bye-pass Jigawa state, 720101, Nigeria; c Department of Plant Sciences, Faculty of Agricultural and Food Sciences, Széchenyi István University, Mosonmagyaróvár, Hungary

**Keywords:** Wastewater, leaching, Nutrient uptake, Nutrient content, Soil bacteria, Choy sum

## Abstract

Washed rice water (WRW) is the leftover water after washing rice grains and is usually discarded. However, WRW contains nutrients leached from rice, making it a potential plant fertilizer. Reusing WRW promotes better water governance, particularly in the face of increased freshwater needs due to population expansion and climate change. Recent experiments in rain shelters have demonstrated the advantages of using WRW as fertilizer. Building on this, our study assessed WRW's efficacy in an open field against NPK fertilizer, both individually and in combination. The treatments were: R3 (3-day fermented WRW), N1 (full recommended NPK rate), N0.5R3 (half NPK rate and R3), and CON (tap water only). These treatments were tested over three consecutive planting cycles of choy sum (*Brassica chinensis* var. *parachinensis*) vegetable. At the end of each planting cycle, measurements were taken for the plant's growth, nutrient content and uptake, as well as various soil chemical properties and bacterial population. Plants were watered daily with 5 mm WRW (R3 and N0.5R3) or tap water (N1 and CON). N0.5R3 showed the best results in terms of plant growth, nutrient content, uptake, and soil nutrient levels. N0.5R3 supplied the most nutrients, especially N, P, and K. Increased plant growth also led to increased plant uptake of nutrients, including micronutrients. Macronutrients had a greater impact on plant biomass than micronutrients, as R3 and N1 had similar results. R3 soils had higher bacterial populations but were more acidic than N1 soils. The negative effect of NPK on bacteria was partially offset by combining NPK with WRW as N0.5R3. No carryover effects were observed, likely because of the high nutrient leaching from heavy rains. These findings confirm WRW's is an effective fertilizer in open fields, but measures like surface mulching are crucial to minimize nutrient leaching prior to its use.

## 1 Introduction

Washed rice water (WRW) is the leftover water from washing rice before cooking. [1,2] revealed that washing rice grains can leach between 7 and 70% of their water-soluble nutrients into the WRW. Given that rice is the world's second most produced cereal and is consumed by over half of the global population [3,4], WRW reuse can potentially conserve substantial amounts of water. The global rice consumption by 2022 is 510 million tons [5]. Assuming 1 kg of rice is washed with 1 L of water, approximately 510 billion L of WRW would be generated and which would typically be discarded into the environment. Reusing this nutrient-enhanced WRW to improve soil fertility and plant growth would thus be beneficial.

WRW reuse should be encouraged, as it is linked to better governance of water [6], especially when freshwater demand is projected to increase by 55% by 2050 [7] owing to population growth and climate change [8,9]. However, most research on WRW reuse has focused on its potential applications in human or animal health and cosmetics, such as skin and hair care, and very little on agricultural use. [6] noted that most studies exploring WRW's agricultural use are published in non-English languages, although a few of them have accompanying English abstracts. Unfortunately, even of the few available studies on WRW for agriculture use have several serious detriments. They often lack scientific rigor, making it difficult to definitively establish WRW's benefits for plants [6]. For instance, many of these studies did not analyze soil nutrient content before and after WRW application [10,11], did not specify WRW application frequencies or the experiment's cultivation area or container size [12,13], and did not compare WRW's effects on plant growth and soil health with those of conventional NPK fertilizers [14,15]. Additionally, they lack comprehensive details for experimental replication and omit crucial follow-up experiments or observations that would substantiate their findings.

Despite their limitations, these studies hint at the potential advantages of WRW. For instance, WRW fermented with cellulolytic bacteria contained N, P, and K nutrients at 0.04, 0.028, and 0.1%, respectively, boosting the growth of pepper plants [16]. [12,17] assessed the impact of WRW alone or mixed with eggshells on eggplant and tomato growth and found significant improvements in crop yield traits, such as plant height, leaf number, and fresh weight, compared to tap water control. [18] further noted that WRW treatment resulted in higher leaf weight and plant height for spinach plants compared to other organic waste treatments, such as animal urine, attributing this to WRW's nutrient content.

Acknowledging the significance of WRW reuse in agriculture and recognizing the limitations of most existing studies, [19] recently conducted a comprehensive and scientifically rigorous investigation into WRW's use as a plant fertilizer. They found WRW contained, on average, in mg L^−1^: 81 N, 16 NO_3_–N, 18.8 NH_4_–N, 1452 S, 65 P, 131 K, 24 Ca, 25 Mg, 0.18 Cu, 0.07 Zn, and 0.12 B. And fermentation of WRW further increased the concentration of most of these nutrients by 7.4–57% and promoted the growth of the following bacteria that could fix N (*Enterobacter ludwigii*, *Pantoea agglomerans*, *Klebsiella pneumoniae*, and *Stenotrophomonas maltophilia*), solubilize P (*Enterobacter ludwigii*, *Enterobacter mori*, *Bacillus velezensis*, *Klebsiella pneumoniae*, *Pantoea agglomerans*, and *Stenotrophomonas maltophilia*), and solubilize K (*Bacillus velezensis*, *Klebsiella pneumoniae*, *Pantoea gglomerans*, and *Stenotrophomonas maltophilia*). These bacteria were identified by gene sequencing. Additionally, indole-3-acetic acid (IAA), a hormone that promotes root growth, was detected in WRW. The bacteria in WRW reached their maximum population after three days of fermentation, after which they decreased in tandem with the reduction in WRW carbon content, their primary food source.

[[19], [20], [21]] further tested the effects of three-day fermented WRW, along with NPK chemical fertilizer, on the growth of two commonly grown leafy vegetables, choy sum (*Brassica chinensis*) and kangkung (*Ipomoea reptans*), under rainshelter conditions. Their trials showed that the benefits of WRW had a carryover effect; that is, its effects intensified over time. Although WRW contains far fewer macronutrients N, P, and K compared to equal weight NPK, WRW either outperformed or matched the NPK in the long run.

During the initial plant growth cycles, the WRW-treated choy sum and kangkung showed lackluster growth, comparable to plants watered only with tap water. NPK fertilizer initially boosted plant growth significantly. However, in the second growth cycle, the WRW-treated choy sum showed comparable or better growth than NPK-treated plants. By the third cycle, WRW-treated kangkung was 50–70% heavier, with a 30% larger total leaf area compared to NPK-treated plants. Additionally, WRW-treated soils showed the highest increase in bacterial population, while NPK-treated soils showed a smaller increase, or in the case of kangkung, a decline.

[2,[19], [20], [21]] concluded that WRW is useful as a plant fertilizer, particularly over the course of several planting cycles, and that WRW is akin to a sustainable liquid biofertilizer [22]. However, their trials were conducted under controlled conditions of a rain shelter. Therefore, this study was a follow-up to their recent work and aimed to evaluate the effects of three-day fermented WRW on choy sum growth under open field conditions. Three treatments were selected: three-day fermented WRW (R3), the full recommended rate of NPK fertilizer (N1), and a combination of half the recommended NPK rate (50%) with R3 (N0.5R3). A control group (CON) with no fertilizer or WRW was also included as a baseline for comparison. The effects of these treatments on choy sum growth, plant nutrient content, and nutrient uptake were evaluated over three consecutive planting cycles. The N0.5R3 treatment was included to test whether the combination of NPK and three-day fermented WRW could further boost yields and soil fertility. We hypothesized that this combined treatment would have the most significant positive effect on choy sum growth over the three planting cycles owing to the complementary effect of NPK and WRW. In short, this study aimed to: 1) determine the effects of the treatments (R3, N0.5R3, N1, and CON) over three planting cycles on choy sum growth, plant nutrient content, and nutrient uptake, and 2) assess the impact of these treatments on various soil chemical properties and bacterial population. Assessments were performed for three planting cycles.

## 2 Materials and methods

### 2.1 Field planting

**Fig. 1:**
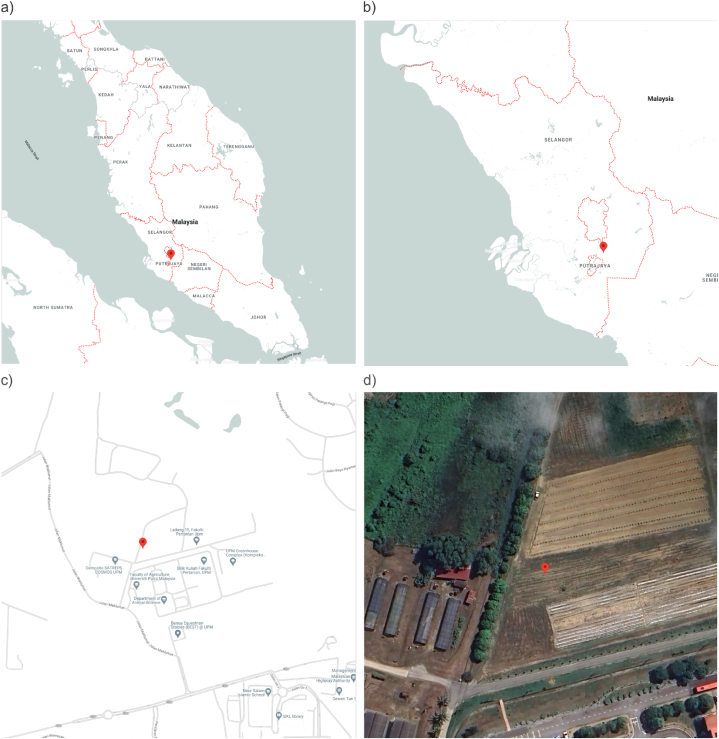
Site of the field experiment: Field No. 15, Faculty of Agriculture, Universiti Putra Malaysia (2.984873° N, 101.734337° E). Site location at the level of: a) country, b) state, and c) town. Aerial view of the plots is (d).

The field experiment was conducted at Field No. 15 (Fig. 1a–d), an experimental field at the Faculty of Agriculture, Universiti Putra Malaysia, Serdang (2.984873° N, 101.734337° E), over three consecutive planting cycles from March to May 2022. The soil had a fine sandy clay texture of the Bungor series (Typic Paleudult), and the initial soil properties are listed in Table 1. The field site had a hot, humid tropical climate, with an average daily air temperature and relative humidity of 28 °C and 85%, respectively, and monthly rainfall between 158 and 492 mm during the field trials.

**Table 1:** Initial mean (±SE) physico-chemical properties of the soil used in the study.

Parameters	Soil properties
pH	4.97 ± 0.16
EC (dS m^−1^)	0.92 ± 0.03
CEC (cmol kg^−1^)	6.14 ± 0.21
	(mg kg^−1^)
Total carbon	10800 ± 400
Organic carbon	9600 ± 78
Total nitrogen	800 ± 63
NH_4_–N	34.54 ± 1.75
NO_3_–N	4.85 ± 0.32
Available P	9.40 ± 0.04
Potassium	176 ± 9
Calcium	210 ± 6
Magnesium	49.21 ± 3.61
Copper	0.12 ± 0.01
Zinc	1.21 ± 0.03
Boron	0.11 ± 0.01
Particle size distribution	(%)
Clay (<2 μm)	35.1 ± 1.05
Silt (2–50 μm)	13.4 ± 0.32
Sand (>50 μm)	51.5 ± 1.72
Texture class (USDA)	Sandy clay
Soil water characteristics	(m^3^ m^−3^)
Saturation	0.67 ± 0.02
Field capacity	0.34 ± 0.01
Permanent wilting point	0.12 ± 0.01

Choy sum (*Brassica chinensis* var. *parachinensis*), a fast-growing leafy vegetable that is widely cultivated and consumed [23], was selected as the test crop. Seeds were germinated in peat moss for ten days, then transplanted into the field with 20 × 20 cm spacing. Following conventional farming practices, the field plots were plowed to a soil depth of 30 cm, followed by rotovation and harrowing. The field plots were protected from the risk of waterlogging or flooding by planting the choy sum on raised beds. Each plot, turned into a raised bed having length × width × height dimensions of 1 × 1 × 0.3 m, was spaced 0.5 m apart, making the total experimental area 42.25 m^2^ (6.5 × 6.5 m). Note that the raised beds were not covered with any surface mulching material. Pre- and post-emergence herbicides were applied for weed control and the insecticide dimethoate was used against pests.

The treatments, with four replications each, comprised: 1) three-day fermented WRW (R3), 2) the full recommended rate of 450 kg ha^−1^ of NPK 15:15:15 fertilizer according to [24] for leafy vegetables (N1), 3) a combination of R3 and half the recommended NPK rate (N0.5R3), and 4) a control group (CON), receiving only tap water. NPK fertilizer was applied evenly to the soil surface prior to field planting in the N1 and N0.5R3 treatments. Daily watering at a rate of 5 mm was performed with tap water for the N1 and CON treatments and with three-day fermented washed rice water for the R3 and N0.5R3 treatments. The treatment plots were monitored for 30 days, after which all the plants were harvested, and the soil was sampled for plant and soil measurements.

The aforementioned process was then repeated for the second and third planting cycles, with the exception that for the subsequent planting cycles, the field plots were left undisturbed (*e.g.*, no plowing, rotorvating, or harrowing again), and the field layout remained unchanged. The same plant and soil parameters measured in the first planting cycle were repeated in the subsequent planting cycles to determine, if any, the carryover or continuous effects of the treatments on the plant and soil properties as well as on the soil microbial population.

### 2.2 Plant and soil parameters

The following plant growth parameters were measured at the end of every planting cycle (30 days after transplant): plant height, number of leaves, shoot fresh and dry weights, and leaf fresh and dry weights. Total leaf area per plant was measured using an LI-3100 Area meter (LI-COR, Nebraska, USA), plant height was measured with a measuring tape, and fresh and dry plant weights were measured using a weighing balance (Multitech, GF-3000, Osaka, Japan). Leaf chlorophyll content was determined using a portable chlorophyll meter (SPAD-502, Minolta Co. Ltd., Osaka, Japan) for a rapid and non-destructive approach that enables in situ measurements. Observations were performed a week after transplanting and then continued weekly. The uppermost and fully expanded leaves were selected for measurement to determine the optimum chlorophyll content. All readings were completed within 1 h to minimize errors caused by the diurnal pattern of photosynthesis [25]. Plant N, C, S content were assayed using CNS Analyzer (LECO Corp., St. Joseph, MI, USA), while plant Ca, Mg, K, Cu, Zn, and B contents were determined by the atomic absorption spectrophotometry (PerkinElmer, PinAAcle 900T, Waltham, MI, USA) after the plant tissues were digested using the dry ashing procedure [26], and plant P was determined using auto analyzer (AA) (Leachat QuikChem FIA+ 8000 series, ON, Canada).

Plant nutrient uptake was calculated by multiplying the total leaf dry weight by its respective nutrient content, and specific leaf area (SLA) was calculated by dividing the total leaf area by the total leaf dry weight.

Soil pH was measured at the end of every planting cycle for pH, NH_4_–N, NO_3_–N, P, K, Ca, Mg, Cu, Zn, and B. Soil pH was measured in a soil-water suspension with a soil:water ratio of 1:2.5 [27] using an 827 pH lab meter (Metrohm AG, Zurich, Switzerland). Soil total C and N were measured by the combustion method [28] using a LECO CR-412 Carbon Determinator (LECO Corp., St. Joseph, MI, USA). Soil organic carbon (SOC), NH_4_–N, and NO_3_–N were determined according to Ref. [29]. The exchangeable base contents (K, Ca, and Mg) and cation exchange capacity (CEC) were assessed using AAS (PinAAcle 900T, PerkinElmer, Waltham, MI, USA) and AA (Leachat QuikChem FIA+ 8000 series, ON, Canada), respectively, after extraction by the leaching method using a neutral 1 M ammonium acetate (NH_4_OAc) solution [30]. Soil P was extracted using the Bray II method [31] and determined using a spectrophotometer (Agilent Technologies 8453 7 Cuvette UV–Vis Spectrophotometer, Boston, MA, USA). Soil particle-size analysis was assessed by the pipetting method [32]. The soil water retention curve for matric potentials of 0.0 to −1.5 MPa was measured by the pressure plate method [33]. Soil total bacterial population was determined at the end of every planting cycle following [34], where 1 g of fresh soil from each experimental unit was sampled and subjected to a series of dilutions of up to 10^8^ and grown on agar plates containing Tryptic Soy Agar by spreading 1 ml of the soil culture. The plates containing the soil culture were incubated for 24 h before enumeration of the bacterial population from each plate (in triplicate) using the colony counting method. Plates with a range of 30–300 colonies were selected and counted as colony-forming units (CFU) per gram of the sample.

### 2.3 Washed rice water

The chemical properties of unfermented WRW, fermented WRW, and tap water (CON) were analyzed. After filtering the WRW (both fermented and unfermented) through a Whatman 1 filter paper (11 μm size), the WRW and tap water were analyzed for pH, EC, total N, NH_4_, NO_3_, C, S, P, K, Ca, Mg, Cu, Zn, and B. Total N, C, and S were analyzed using a CNS analyzer (LECO Corp., St. Joseph, MI, USA); K, Ca, Mg, Cu, Zn, and B were analyzed using atomic absorption spectrophotometer (AAS) (PerkinElmer, PinAAcle, 900T, Waltham, MI, USA); and P was determined using Auto Analyzer (AA) (Leachat QuikChem FIA+ 8000 series, ON, Canada). The contents of NH_4_–N and NO_3_–N in both R3 and tap water were determined using the Kjeldahl procedure [29], while the pH and EC were measured using an 827 pH and EC lab meter (Metrohm AG, Zurich, Switzerland) [27].

### 2.4 Statistical analysis

The field was laid out as a Randomized Complete Block (RCB) design, with each of the four treatments replicated four times. Given the focus of this study was also on examining the potential carryover effects of treatments over successive planting cycles, a split-plot approach was adopted. In this design, the treatments served as the main plots (or whole-plot factors), while the planting cycles, which were of particular interest because of their potential interactions with the treatments, were treated as subplots (or the smallest experimental units). This design inherently emphasizes the interactions between the treatments and planting cycles by allowing all levels of the subplot (planting cycle) to be tested within each whole plot level (treatment). To analyze the data, and considering that the same parameters were repeatedly measured in every planting cycle in the same plots, a pooled ANOVA was employed, following a repeated-measures RCB design [35]. ANOVA was by Minitab (version 20) software package (Pennsylvania State University, State College, PA, USA). Main effects and interaction plots were generated using OriginPro software version 2021 (OriginLab Corporation, Northampton, MA, USA). Significant treatment means were separated using the Bonferroni test at a 5% level of significance.

## 3 Results

### 3.1 Nutrients added

The nutrient content in the unfermented WRW (R0), three-day fermented WRW (R3), and tap water are shown in Table 2, and the cumulative amounts of nutrients added by the various treatments over the three planting cycles (totalling 90 days) are shown in Table 3. Fermenting WRW over three days increased its nutrient levels by 7.4–56% compared with R0. Compared with R0, the soil pH in R3 declined because of the formation of organic acids during fermentation [36]. Total C in R3 also declined compared to R0 because C is the major energy source for bacteria [37], which would decline as the bacteria proliferated in the WRW during fermentation [36,37]. This was evident in the increase in the soil bacterial population in R3 compared to that in R0. As expected, tap water had the lowest amount of nutrients, as it was treated according to the National Water Quality Standards for Malaysia [38] for safe human consumption.

**Table 2:** Means (±SE) element analyses of unfermented and fermented tap water and washed rice water (WRW). All units are in mg kg^−1^ unless otherwise indicated.

Parameters	Unfermented WRW	R3	Tap Water
pH	6.53 ± 0.02	4.53 ± 0.08	6.58 ± 0.02
EC (μS cm^−1^)	285.83 ± 34.53	551.30 ± 21.42	125.36 ± 28.21
Total C	2850.23 ± 120.74	2160.43 ± 401.23	30.02 ± 2.23
S	110.10 ± 40.39	120.62 ± 10.57	100.21 ± 10.31
Total N	160.11 ± 5.20	220.11 ± 41.62	30.20 ± 4.12
NH_4_–N	10.50 ± 1.68	11.80 ± 0.36	1.44 ± 0.04
NO_3_–N	5.48 ± 1.41	5.40 ± 0.06	1.45 ± 0.03
P	90.92 ± 3.76	209.81 ± 11.21	0.05 ± 0.02
K	118.11 ± 14.21	135.80 ± 9.22	5.74 ± 0.15
Ca	8.52 ± 2.10	13.61 ± 0.53	10.95 ± 0.06
Mg	27.9 ± 1.76	66.81 ± 3.22	0.97 ± 0.06
Cu	0.19 ± 0.01	0.21 ± 0.03	trace
Zn	0.07 ± 0.01	0.25 ± 0.01	trace
B	0.12 ± 0.02	0.18 ± 0.02	trace
Bacterial population (CFU mL^−1^)	1.12 × 10^2^ ± 10^1^	2.12 × 10^8^ ± 10^3^	–

Note: − is not detected; R3 is washed rice water fermented for three days; CFU is colony-forming units.

**Table 3:** Mean (±SE) total nutrients added by different treatments over the three planting cycles (90 days in total). Units are in grams, unless otherwise indicated.

Parameters	R3	N1	N0.5R3	CON
Total C	90.72 ± 2.70	–	90.72 ± 2.70	1.320 ± 0.06
S	5.06 ± 0.21	–	5.06 ± 0.21	1.130 ± 0.16
Total N	8.24 ± 0.46	7.83 ± 0.26	13.07 ± 0.61	1.131 ± 0.08
NH_4_–N	0.50 ± 0.07	–	0.50 ± 0.07	0.091 ± 0.01
NO_3_–N	0.22 ± 0.06	–	0.22 ± 0.06	0.090 ± 0.01
P	7.80 ± 0.32	5.73 ± 0.28	11.53 ± 0.46	0.031 ± 0.01
K	5.70 ± 0.18	6.61 ± 0.16	9.31 ± 0.30	0.361 ± 0.02
Ca	0.56 ± 0.03	–	0.56 ± 0.03	0.689 ± 0.04
Mg	2.80 ± 0.11	–	2.80 ± 0.11	0.061 ± 0.01
Cu (mg)	8.66 ± 0.31	–	8.66 ± 0.31	0.151 ± 0.01
Zn (mg)	10.64 ± 0.43	–	10.64 ± 0.43	0.333 ± 0.01
B (mg)	7.62 ± 0.21	–	7.62 ± 0.21	0.175 ± 0.01

Note: R3 is fermented WRW for three days; N1 is the full rate of 450 kg ha^−1^ NPK 15:15:15; N0.5R3 is the combination of 50% of the recommended NPK rate and R3; CON is tap water; and – is not determined.

### 3.2 Plant growth

**Fig. 2:**
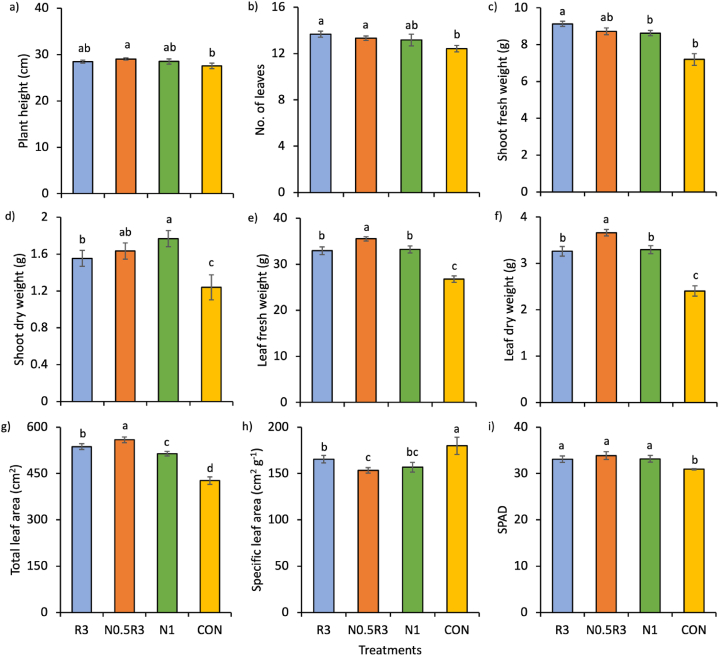
Effects of treatments on: a) plant height, b) number of leaves, c) shoot fresh weight, d) shoot dry weight, e) leaf fresh weight, f) leaf dry weight, g) total leaf area, h) specific leaf area, and i) SPAD. In the same chart, means with different letters differ significantly from one another according to Bonferroni's test at the 5% level. Means (±SE) were taken per 3 plants for shoot and leaf fresh and dry weight, total, and specific leaf area.

The interaction between the treatments and planting cycles did not significantly affect plant growth parameters (p > 0.05). However, the main effect of the treatments was significant (p < 0.01) (Fig. 2), with N0.5R3 treatment producing the highest leaf weights (fresh and dry), and together with R3, the highest total leaf area (Fig. 2e–g). The WRW-based treatments (R3 and N0.5R3) and N1 produced similar plant growth (p > 0.05) in terms of plant height, number of leaves, shoot weights (fresh and dry), specific leaf area (SLA), and SPAD (Fig. 2a–d, h, and i). Interestingly, CON had the highest specific leaf area by 17% over the others (p < 0.01) (Fig. 2h).

### 3.3 Plant nutrient content

**Fig. 3:**
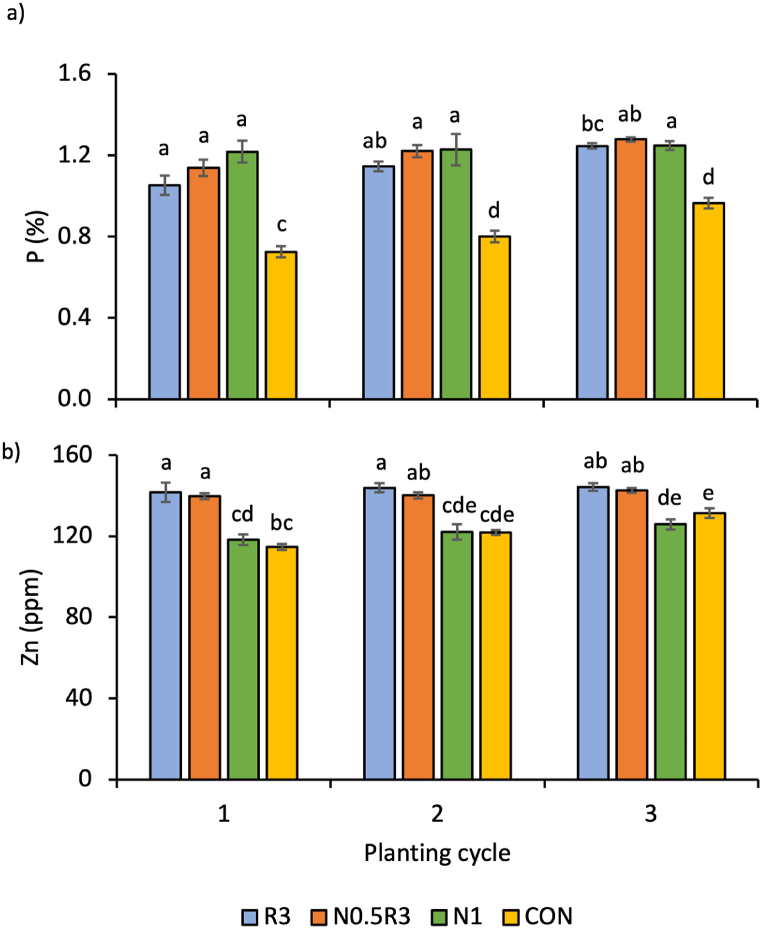
Mean (±SE) of plant: a) P and b) Zn content due to the interaction between treatments and planting cycles. In the same chart, means with different letters differ significantly from one another according to Bonferroni's test at the 5% level. Pairwise comparisons were performed across all the planting cycles and treatments. Note: R3 is fermented washed rice water for three days; N1 is the full rate of 450 kg ha^−1^ NPK 15:15:15; N0.5R3 is the combination of 50% of the recommended NPK rate and R3; and CON is tap water.

There was a significant interaction (p < 0.01) between the treatments and planting cycles, but only for plant P and Zn contents (Fig. 3). Zn levels in plants were the highest in the WRW-based treatments (N0.5R3 and R3) (Fig. 3b), whereas plant P levels were generally similar across all treatments, except for CON, where it was the lowest (Fig. 3a).

**Fig. 4:**
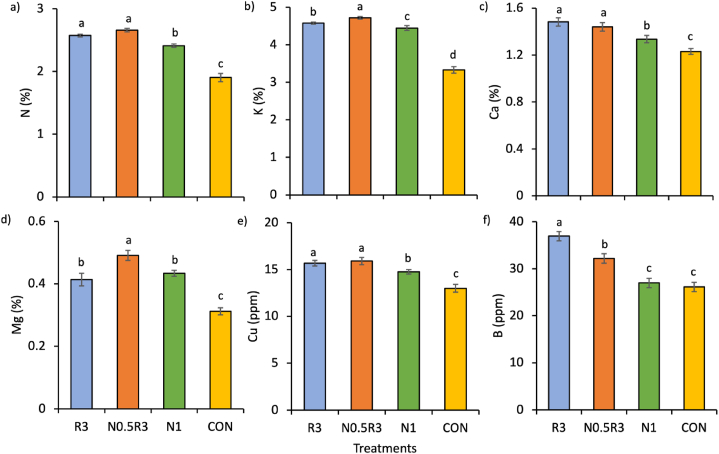
Mean (±SE) of plant: a) N, b) K, c) Ca, d) Mg, e) Cu, and f) B as influenced by different treatments. In the same chart, means with different letters differ significantly from one another according to Bonferroni's test at the 5% level. Note: R3 is fermented washed rice water for three days; N1 is the full rate of 450 kg ha^−1^ NPK 15:15:15; N0.5R3 is the combination of 50% of the recommended NPK rate and R3; and CON is tap water.

The treatments had a significant main effect (p < 0.05) on the content of other nutrients in the plants (Fig. 4). N0.5R3 produced plants with the highest levels of K and Mg (Fig. 4b and d), whereas, together with R3, the highest N, Ca, and Cu concentrations (Fig. 4a, c, and e). The plant nutrient levels in WRW-based treatments were higher by 6.1–13.4% than in N1. Plant B content was the highest in R3, followed by N0.5R3 and N1 (Fig. 4f). The CON treatment consistently resulted in the lowest content of all plant nutrients, including P and Zn.

### 3.4 Plant nutrient uptake

**Fig. 5:**
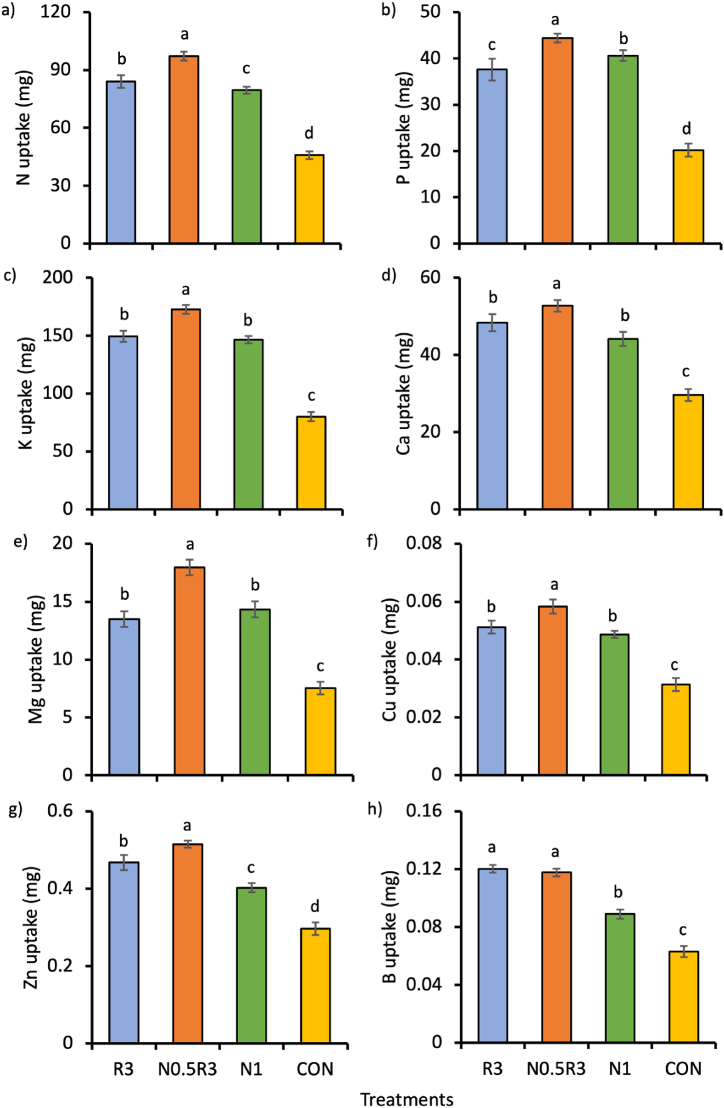
Mean (±SE) of plant nutrient uptake of: a) N, b) P, c) K, d) Ca, e) Mg, f) Cu, g) Zn, and h) B, as influenced by different treatments. In the same chart, means with different letters differ significantly from one another according to Bonferroni's test at the 5% level. Note: R3 is fermented washed rice water for three days; N1 is the full rate of 450 kg ha^−1^ NPK 15:15:15; N0.5R3 is the combination of 50% of the recommended NPK rate and R3; and CON is tap water.

The main effect of the treatments significantly influenced plant nutrient uptake but not the interaction between the treatments and planting cycles. Plants in the N0.5R3 plots had the highest uptake of all nutrients (Fig. 5a–g), except for B, which had the highest uptake compared to those in the R3 plots (Fig. 5h). Compared to other treatments, N0.5R3 increased plant nutrient uptake in the following extends: N increased by 16–112%; P by 9–120%; K by 16–116%; Ca by 9–78%; Mg by 25–139%; Cu by 13–87%; and Zn by 10–74% (Fig. 5a–g). For most nutrients, the uptake in N0.5R3 was at least 100 times higher than that in CON.

### 3.5 Soil properties

**Fig. 6:**
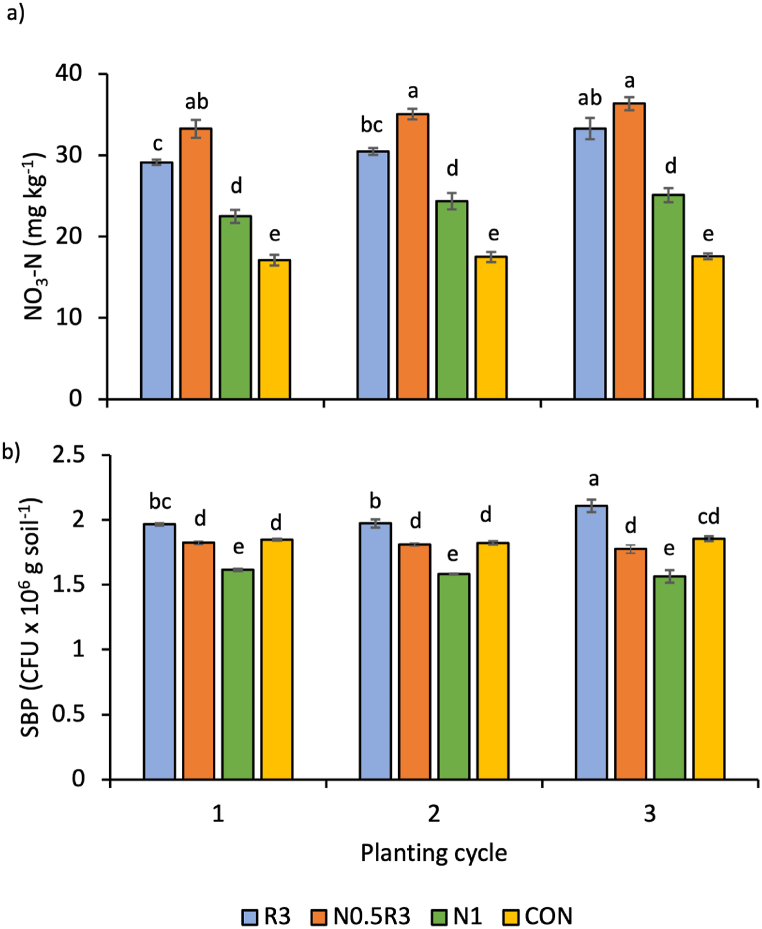
Mean (±SE) of soil: a) NO_3_–N and b) soil bacterial population (SBP) due to the interaction between treatments and planting cycle. In the same chart, means with different letters differ significantly from one another according to Bonferroni's test at the 5% level. Pairwise comparisons were performed across all planting cycles and treatments. Note: R3 is fermented washed rice water for three days; N1 is the full rate of 450 kg ha^−1^ NPK 15:15:15; N0.5R3 is the combination of 50% of the recommended NPK rate and R3; and CON is tap water.

The interaction between treatments and planting cycles significantly affected only soil NO_3_–N and soil bacterial populations (p < 0.01) (Fig. 6). For every planting cycle, soil NO_3_–N was highest in N0.5R3, followed by R3, N1, and CON (Fig. 6a). Soil NO_3_–N in the N0.5R3 plots was 9–107% higher than that in the other treatment plots. However, the levels of soil NO_3_–N did not increase with the progression of the planting cycles.

In the third planting cycle, the soil bacterial population was the highest for R3, at 2.11 × 10^6^ CFU g^−1^ soil (p < 0.01) (Fig. 6b). At every planting cycle, R3 had the highest soil bacterial population, followed by CON and N0.5R3, both of which had comparable populations, and N1 had the lowest bacterial count. The soil bacterial population also did not increase with the progression of planting cycles.

**Fig. 7:**
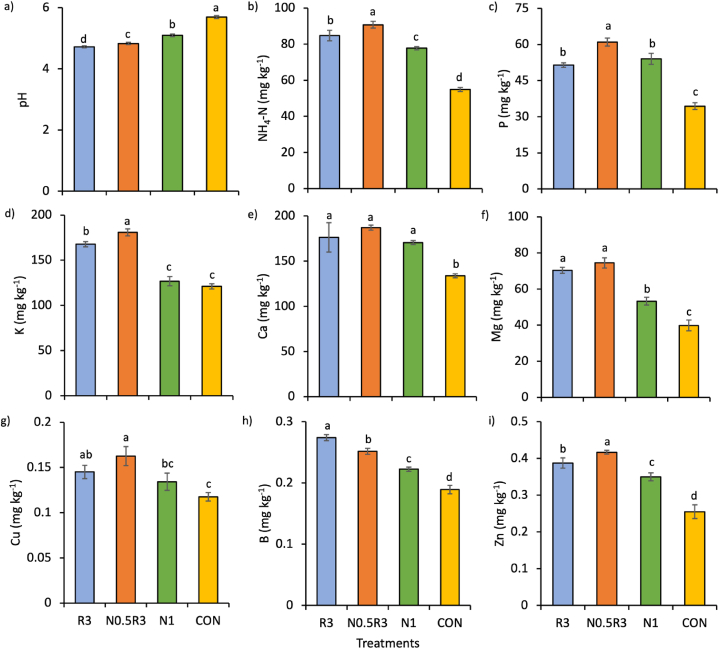
Mean (±SE) of treatment effects on soil properties: a) pH, b) NH_4_–N, c) P, d) K, e) Ca, f) Mg, g) Cu, h) B, and i) Zn content after harvest as influenced by different treatments. In the same chart, means with different letters differ significantly from one another according to Bonferroni's test at the 5% level. Note: R3 is fermented washed rice water for three days; N1 is the full rate of 450 kg ha^−1^ NPK 15:15:15; N0.5R3 is the combination of 50% of the recommended NPK rate and R3; and CON is tap water.

The main effect of the treatments was significant on all soil properties, except for total N (Fig. 7). Soil pH in the CON plots was 10–19% higher (less acidic) than that in the other treatments (p < 0.01) (Fig. 7a). Soil pH was the highest in CON, followed by N1, N0.5R3, and R3, with the latter being the most acidic at pH 4.7. The soils in the N0.5R3 plots had the highest levels of NH_4_–N, P, K, and Zn (Fig. 7b–d and i). The soil Ca, Mg, and Cu levels in the WRW-based treatments were generally similar to or higher than those of N1 (Fig. 7e–g). The soils in the CON plots had the lowest nutrient content.

## 4 Discussion

The WRW-based treatments had either higher or similar plant growth, plant nutrient content, plant nutrient uptake, and soil nutrient content than N1. Notably, the N0.5R3 treatment, a combination of NPK and WRW, resulted in plants with the highest total leaf fresh and dry weights, and, along with the R3 treatment, yielded the highest total leaf area (Fig. 2e–g). Additionally, plants treated with N0.5R3 had the highest nutrient uptake for all nutrients except for B (Fig. 5), where both N0.5R3 and R3 shared the highest B uptake (Fig. 5h). The N0.5R3 plants also contained the highest levels of K and Mg (Fig. 4b and d). Although total soil N did not differ significantly across treatments, the differences in available N (NO_3_–N and NH_4_–N) were significant, being the highest in the N0.5R3 plots, along with P, K, and Zn (Figs. 6a, 7b-d, and 7i).

Over the 90-day duration of field trials, the N0.5R3 treatment, despite having only half the NPK rate as compared to N1, still introduced a greater quantity of nutrients into each experimental unit owing to the contribution from its R3 component. This treatment combination supplied 59–70%, 47–92%, and 41–63% more N, P, and K, respectively, than the sole applications of N1 and R3 (Table 3).

The greater supply of N, P, and K nutrients may explain why plant growth and nutrient uptake were the highest in the N0.5R3 treatment plots. Due to interactions between nutrients [39,40], the addition of one or more nutrients can lead not only to higher plant growth but also to higher plant uptake of other nutrients, including micronutrients, such as observed by [[41], [42], [43], [44]]. For instance, [43] observed that the application of N fertilizer enhanced maize uptake for non-N nutrients, such as P, S, Cu, and Zn, by up to 420%. Similarly, [44] observed that N fertilization in a forage legume increased both plant growth and nutrient uptake of K, Ca, Mg, Na, Fe, and Cu. [44] attributed the increased plant nutrient uptake to increased root growth (in terms of root mass and length), and thus increasing access of the legume to nutrients in the soil.

R3 and N1 treatments produced similar results for plant growth. This is likely because both supplied similar amounts of N, P, and K nutrients over the 90-day period (Table 3), even though R3 additionally provided micronutrients that were not present in N1. This suggests that the macronutrients N, P, and K had a more influential and direct role in increasing the plant biomass of choy sum than micronutrients, as observed in the N0.5R3 treatment.

All macro- and micronutrients are essential for plant growth, and each serves multiple functions that contribute to the overall growth of the plant. Nutrient N is typically linked to the development of leaves and stems because of its pivotal role in photosynthesis and because it is a key component of chlorophyll. Nutrient P, on the other hand, is generally associated with root development, whereas nutrient K, with its integral role in a wide range of plant processes such as enzyme activation, photosynthesis, water use efficiency, and protein synthesis, is often associated with the overall health of the plant [[45], [46], [47], [48]]. Furthermore, [49] observed that an association between micronutrients and biomass was only evident in grasslands when their N and P demands were satisfied. However, in grasslands limited by N and P, this link between micronutrients and grass biomass was absent. [49] concluded that their observations were aligned with the concept of serial co-limitation, in that the grasslands only showed a response to added micronutrients after the supplementation of macronutrients N and P was adequate.

However, soils in the R3 plots were different from those in N1. Compared to N1, R3-treated soils had a higher soil bacterial population. Despite producing comparable plant growth with R3, the sole application of NPK fertilizer in the N1 treatment had a detrimental impact on the soil bacterial population, lowering it to even smaller than that of the control. In contrast, repeated application of WRW in the R3 treatment boosted the soil bacterial population to the highest level compared with all other treatments. However, combining WRW with NPK in the N0.5R3 treatment reduced the detrimental impact of NPK to have comparable soil bacterial populations to CON, although it was still lower than that in R3. Soils in R3 plots were also more acidic than those in all other treatments. As observed by [36,50,51], WRW ferments over time, and the longer the fermentation time, the lower the pH because of the increased formation of organic acids by the microbes. Despite these differences, neither the soil bacterial population nor soil pH changed with the planting cycles for all treatments, including R3 and N1. As a result, they did not seem to be as crucial or influential as other factors affecting plant growth, at least within the three planting cycles.

CON, as expected, performed the worst on plant growth and had the lowest plant nutrient content and uptake because it supplied the least amount of nutrients, which were lower by 400–750% than the other treatments (Table 2, Table 3). Plants in the CON plots, however, had the highest specific leaf area (SLA), which was calculated as the ratio of total leaf area to total leaf dry weight. SLA is sometimes used as a proxy for overall plant growth [52], but its relationship with plant growth has not always been observed. [53], for instance, conducted fertilizer trials across four continents and observed leaf nutrient content, not SLA, that responded to four years of nutrient addition. SLA is sometimes also regarded as leaf thickness, such that thin leaves would have a higher SLA than thick leaves. In this context, thicker leaves (low SLA) in rice and several other C3 trees have been observed by [54,55] to have higher N and chlorophyll content, along with a higher concentration of photosynthetic enzymes per unit leaf area. Regardless, it remains evident in this study that plants in the CON plots fared the worst in nearly all measured plant and soil parameters because of the very low nutrient levels supplied only through tap water.

The ANOVA results highlighted that the primary influence on most of the measured parameters was the main effect of the treatments rather than the interaction between treatments and planting cycles. Plant growth metrics such as plant height, number of leaves, shoot fresh and dry weights, leaf fresh and dry weights, total leaf area, specific leaf area, and SPAD were significantly affected only by the main effect of the treatments. This indicates that the WRW-based treatments did not demonstrate carryover effects, where the residual benefits from the fertilizer persisted from one planting cycle to another. No consistent pattern was observed in terms of increasing plant growth, nutrient content, nutrient uptake, or soil nutrient content in the subsequent planting cycles. These observations are in contrast to those reported by [19,21], who observed that the effects of WRW on the growth of choy sum and kangkung had amplified over time. During the first planting cycle, WRW-treated plants experienced lackluster growth that was only slightly or no better than that of the control plants that received only tap water. However, from the second planting cycle for the choy sum and the third for kangkung, plants treated with WRW exhibited growth comparable to or higher than those treated with NPK. Moreover, soils that received the WRW treatment showed a progressive increase in bacterial populations with each subsequent planting cycle. Nonetheless, their experiments were conducted under a rain shelter, where the water supply was strictly controlled. In contrast, this study was carried out in an open field and was subjected to unpredictable fluctuations in weather conditions, particularly rainfall.

In the present study, rainfall likely led to an increase in nutrient loss through leaching. The field trials conducted in March, April, and May 2022 experienced monthly rainfall amounts of 492, 156, and 256 mm, respectively. The high rainfall, especially in March, would have likely exacerbated the leaching process, and WRW, owing to its liquid state, would be very vulnerable to these losses, especially in soils that have become very wet or saturated from heavy rainfall. Our trial plots were only protected against waterlogging or flood conditions by planting the choy sum on raised beds. The plots were not covered with any materials. Moreover, the soil texture at this study site was sandy clay with 52% sand and 35% clay (Table 1). [56] found that the extent of leaching loss from WRW depended on soil texture. Applying 5 mm of WRW daily to a sandy clay loam soil (72% sand; 22% clay) over eight weeks resulted in 13–20% more leaching volume and 21–47% higher nutrient losses, particularly K (due to its high mobility in soils), than silt loam (28% sand; 18% clay) and clay (26% sand; 65% clay) soils.

Soils with coarser textures, owing to their larger pore sizes, are therefore prone to greater water loss through percolation than finer-textured soils. As a result, it is probable that in this study, WRW experienced substantial leaching losses due to rainfall. These losses may have been significant enough to prevent the carryover of any residual fertilizer benefits into the subsequent planting cycles. Our results revealed the risk of applying liquid fertilizers, such as WRW, to unprotected soil surfaces in open fields. Therefore, it is important to take preventive measures against high nutrient loss through leaching, particularly in coarse-textured soils, before applying WRW. One such strategy is the application of surface mulches.

[6] has conducted an extensive review of previous WRW studies on its use as a plant fertilizer. The majority of these studies, whether using WRW alone or in combination with other wastewater or organic materials, have focused on short-term crops, primarily vegetables, such as *Brassica* spp., *Lycopersicon esculentum*, *Solanum melongena*, *Adenium obesum*, *Capsicum* spp., and *Spinacia oleracea*. Most research has reported positive effects of WRW on crops, noting outcomes such as an increase in leaf count, increased plant biomass, larger stems, higher chlorophyll levels, and faster plant growth. However, as stated earlier, [6] pointed out that almost all prior WRW research had several deficiencies. For instance, they did not report the initial and final soil physicochemical properties or nutrient uptake of plants. This omission complicates the process of comparing our findings with those of previous WRW studies, especially regarding soil bacterial populations, a parameter seemingly overlooked in other studies. Only the most recent investigations [[19], [20], [21],^36^,^37^,^56^] have sought to rectify these deficiencies by undertaking a more methodical approach to WRW research. Yet, the results from these studies were derived from laboratory and rainshelter settings. The present study extends these investigations by assessing the WRW under open-field conditions.

This study demonstrated that WRW-based treatments either increased or maintained similar levels of plant growth, nutrient content, and uptake as the N1 treatment, which is in agreement with the results obtained under rain shelter conditions by [19,21]. Notably, this study tested the combined effects of NPK and R3 (N0.5R3), and their combination resulted in the most favorable outcomes for plant growth and nutrient uptake. However, despite the benefits of WRW, this study also highlighted potential challenges such as the lack of observed carryover effects, likely owing to the vulnerability of liquid WRW to leaching, especially in coarse-textured soils, as observed by [56]. The findings of this study underscore the importance of considering environmental factors and soil texture when applying liquid fertilizers such as WRW, and suggest that preventive measures, such as surface mulches, would be beneficial for mitigating nutrient leaching losses.

## 5 Conclusions

This study aimed to evaluate the effects of various treatments (R3, N0.5R3, N1, and CON) over three planting cycles on choy sum growth, plant nutrient content, and nutrient uptake and to understand the impact of these treatments on soil chemical properties and bacterial populations.

From these results, it is evident that the WRW-based treatments, particularly the N0.5R3 treatment, either enhanced or maintained comparable levels of choy sum growth, nutrient content, and uptake relative to the N1 treatment. Specifically, the N0.5R3 treatment, which combined NPK and WRW, showed the most favorable outcomes in terms of plant growth and nutrient uptake. This treatment, despite having only half the NPK rate as N1, introduced a greater quantity of nutrients into each experimental unit owing to the contribution from its R3 component. This suggests a synergistic effect when NPK is combined with WRW, leading to better plant growth and uptake of nutrients, including micronutrients.

There were notable differences between the treatments in terms of soil chemical properties and bacterial populations. Soils treated with R3 had a higher bacterial population compared to those treated with N1. The sole application of NPK fertilizer in the N1 treatment negatively affected the soil bacterial population, reducing it even lower than in the control (CON). However, the combination of WRW and NPK in the N0.5R3 treatment partially moderated the detrimental impact of NPK, resulting in soil bacterial populations comparable to those in CON, albeit still lower than those in R3. Additionally, the R3-treated soils were more acidic than those in the other treatments, likely because of the fermentation process of WRW over time.

WRW-based treatments exerted no carryover effects across planting cycles. ANOVA showed the interaction effect between treatments and planting cycles were non-significant on most parameters. This differs from recent studies that found WRW effects increased over time. Leaching of liquid WRW, especially in coarse-textured soils and high rainfall, may explain the disparity in this study. This underscores the need to consider environmental factors and soil texture when using liquid fertilizers, such as WRW. WRW-based treatments, especially NPK and R3, improved plant growth and nutrient uptake, but leaching risks must be addressed in open field conditions.

## Funding statement

This research was funded by Uni. Putra Malaysia Grant 2022, Geran Inisiatif Putra Siswazah (GP-10.13039/501100003782IPS) (No. GP-IPS/2022/9709600).

## Author contribution statement

Christopher Boon Sung Teh: Conceived and designed the experiments; Performed the experiments; Analyzed and interpreted the data; Contributed reagents, materials, analysis tools or data; Wrote the paper.

Abba Nabayi: Conceived and designed the experiments; Performed the experiments; Analyzed and interpreted the data; Wrote the paper.

Kee Zuan Ali Tan: Ngai Paing Tan: Conceived and designed the experiments; Analyzed and interpreted the data; Contributed reagents, materials, analysis tools or data.

Dora Beke: Analyzed and interpreted the data.

## Data availability statement

Data will be made available on request.

## Declaration of competing interest

The authors declare that they have no known competing financial interests or personal relationships that could have appeared to influence the work reported in this paper.
