# Supplementary material for: Combined benefits of fermented washed rice water and NPK mineral fertilizer on plant growth and soil fertility over three field planting cycles
Source: Heliyon. 2023 Sep 15;9(9):e20213. doi: 10.1016/j.heliyon.2023.e20213 (PMC10559983; doi:10.1016/j.heliyon.2023.e20213)
Supplement: Multimedia component 1 [file mmc1.docx]

Table A1. Means (±SE) of plant growth parameters due to the four treatments: R3 (three-day fermented washed rice water), N1 (recommended rate of NPK 15:15:15 fertilizer at 450 kg ha^-1^), N0.5R3 (combined half the recommended rate of NPK and R3), and CON (control or tap water).

| **TRT** | **CYCLE** | **PLH** | **LN** | **SFW** | **SDW** | **LFW** | **LDW** | **LA** | **SLA** | **SPAD** |
| --- | --- | --- | --- | --- | --- | --- | --- | --- | --- | --- |
| N1 | 1 | 28.35 ± 0.41 | 13.00 ± 0.58 | 8.33 ± 0.14 | 1.65 ± 0.03 | 32.83 ± 0.79 | 3.22 ± 0.04 | 504.50 ± 9.43 | 157.03 ± 3.88 | 33.00 ± 0.77 |
| N0.5R3 |  | 28.88 ± 0.21 | 13.00 ± 0.00 | 8.42 ± 0.20 | 1.52 ± 0.07 | 35.13 ± 0.43 | 3.58 ± 0.02 | 570.25 ± 10.35 | 159.42 ± 3.19 | 33.70 ± 0.79 |
| R3 |  | 28.33 ± 0.32 | 13.50 ± 0.29 | 8.83 ± 0.13 | 1.44 ± 0.03 | 32.53 ± 0.83 | 3.18 ± 0.08 | 540.75 ± 10.14 | 170.19 ± 2.62 | 32.90 ± 0.78 |
| CON |  | 27.43 ± 0.71 | 12.25 ± 0.25 | 6.90 ± 0.18 | 1.12 ± 0.07 | 26.38 ± 0.70 | 2.32 ± 0.08 | 414.25 ± 13.81 | 178.54 ± 4.26 | 30.75 ± 0.19 |
| N1 | 2 | 28.10 ± 0.67 | 13.25 ± 0.48 | 8.23 ± 0.14 | 1.63 ± 0.12 | 32.70 ± 0.73 | 3.09 ± 0.11 | 508.75 ± 6.75 | 165.38 ± 7.25 | 33.05 ± 0.73 |
| N0.5R3 |  | 28.62 ± 0.25 | 13.50 ± 0.29 | 8.32 ± 0.17 | 1.49 ± 0.09 | 35.00 ± 0.46 | 3.45 ± 0.10 | 549.50 ± 8.84 | 159.43 ± 4.01 | 33.75 ± 0.83 |
| R3 |  | 28.07 ± 0.34 | 13.75 ± 0.25 | 8.73 ± 0.15 | 1.41 ± 0.12 | 32.40 ± 0.81 | 3.06 ± 0.12 | 525.50 ± 9.26 | 172.50 ± 4.65 | 32.95 ± 0.66 |
| CON |  | 27.17 ± 0.54 | 12.50 ± 0.29 | 6.80 ± 0.39 | 1.10 ± 0.17 | 26.25 ± 0.72 | 2.20 ± 0.13 | 433.25 ± 15.79 | 199.55 ± 15.08 | 30.80 ± 0.12 |
| N1 | 3 | 29.10 ± 0.67 | 13.25 ± 0.48 | 9.33 ± 0.14 | 2.03 ± 0.12 | 34.20 ± 0.73 | 3.59 ± 0.11 | 528.75 ± 4.97 | 147.71 ± 4.93 | 33.45 ± 0.73 |
| N0.5R3 |  | 29.62 ± 0.25 | 13.50 ± 0.29 | 9.42 ± 0.17 | 1.89 ± 0.09 | 36.50 ± 0.46 | 3.95 ± 0.10 | 557.00 ± 9.09 | 141.02 ± 1.49 | 34.15 ± 0.83 |
| R3 |  | 29.07 ± 0.34 | 13.75 ± 0.25 | 9.83 ± 0.15 | 1.81 ± 0.12 | 33.90 ± 0.81 | 3.56 ± 0.12 | 544.25 ± 8.13 | 153.51 ± 4.81 | 33.35 ± 0.66 |
| CON |  | 28.17 ± 0.54 | 12.50 ± 0.29 | 7.90 ± 0.39 | 1.50 ± 0.17 | 27.75 ± 0.72 | 2.70 ± 0.13 | 433.00 ± 6.67 | 161.67 ± 8.41 | 31.20 ± 0.12 |

PLH – plant height (cm); LN – leaf number; SFW and SDW – shoot fresh and dry weights (g); LFW and LDW – leaf fresh and dry weights (g); LA – total leaf area (cm^2^); SLA – specific leaf area (cm^2^ g^-1^); SPAD – chlorophyll recording

Table A2. Means (±SE) of plant nutrient content due to the four treatments: R3 (three-day fermented washed rice water), N1 (recommended rate of NPK 15:15:15 fertilizer at 450 kg ha^-1^), N0.5R3 (combined half the recommended rate of NPK and R3), and CON (control or tap water). All units are in % except for Cu, Zn, and B which are in ppm.

| **TRT** | **CYCLE** | **N** | **P** | **K** | **Ca** | **Mg** | **Cu** | **Zn** | **B** |
| --- | --- | --- | --- | --- | --- | --- | --- | --- | --- |
| N1 | 1 | 2.40 ± 0.03 | 1.22 ± 0.05 | 4.38 ± 0.07 | 1.29 ± 0.03 | 0.43 ± 0.01 | 14.65 ± 0.23 | 118.25 ± 2.59 | 26.70 ± 0.40 |
| N0.5R3 |  | 2.69 ± 0.02 | 1.14 ± 0.04 | 4.77 ± 0.03 | 1.42 ± 0.02 | 0.49 ± 0.01 | 15.68 ± 0.34 | 139.73 ± 1.44 | 32.30 ± 0.53 |
| R3 |  | 2.58 ± 0.02 | 1.05 ± 0.05 | 4.59 ± 0.02 | 1.51 ± 0.03 | 0.40 ± 0.02 | 14.45 ± 0.22 | 141.58 ± 4.76 | 38.68 ± 0.92 |
| CON |  | 1.81 ± 0.07 | 0.73 ± 0.03 | 3.25 ± 0.06 | 1.20 ± 0.01 | 0.30 ± 0.01 | 11.88 ± 0.83 | 114.58 ± 1.44 | 25.05 ± 0.33 |
| N1 | 2 | 2.41 ± 0.02 | 1.23 ± 0.02 | 4.45 ± 0.06 | 1.34 ± 0.03 | 0.44 ± 0.02 | 14.75 ± 0.21 | 122.05 ± 3.81 | 26.75 ± 1.16 |
| N0.5R3 |  | 2.68 ± 0.04 | 1.22 ± 0.03 | 4.71 ± 0.02 | 1.45 ± 0.04 | 0.50 ± 0.02 | 15.88 ± 0.37 | 142.60 ± 1.56 | 32.25 ± 0.46 |
| R3 |  | 2.59 ± 0.02 | 1.15 ± 0.08 | 4.56 ± 0.03 | 1.49 ± 0.04 | 0.41 ± 0.02 | 16.10 ± 0.29 | 143.85 ± 2.22 | 36.60 ± 1.32 |
| CON |  | 1.88 ± 0.08 | 0.80 ± 0.03 | 3.38 ± 0.09 | 1.25 ± 0.04 | 0.32 ± 0.01 | 13.55 ± 0.22 | 121.80 ± 1.13 | 26.13 ± 0.64 |
| N1 | 3 | 2.42 ± 0.03 | 1.25 ± 0.01 | 4.50 ± 0.06 | 1.37 ± 0.03 | 0.44 ± 0.01 | 14.85 ± 0.28 | 125.80 ± 2.52 | 27.45 ± 1.18 |
| N0.5R3 |  | 2.61 ± 0.02 | 1.28 ± 0.01 | 4.68 ± 0.05 | 1.46 ± 0.05 | 0.49 ± 0.02 | 16.15 ± 0.47 | 140.18 ± 1.25 | 32.03 ± 0.68 |
| R3 |  | 2.55 ± 0.03 | 1.25 ± 0.02 | 4.58 ± 0.02 | 1.46 ± 0.04 | 0.43 ± 0.01 | 16.43 ± 0.37 | 144.30 ± 1.93 | 35.58 ± 0.51 |
| CON |  | 2.03 ± 0.06 | 0.97 ± 0.03 | 3.36 ± 0.12 | 1.25 ± 0.02 | 0.32 ± 0.01 | 13.53 ± 0.19 | 131.40 ± 2.31 | 27.15 ± 0.95 |

Table A3. Means (±SE) of plant nutrient uptake due to the four treatments: R3 (three-day fermented washed rice water), N1 (recommended rate of NPK 15:15:15 fertilizer at 450 kg ha^-1^), N0.5R3 (combined half the recommended rate of NPK and R3), and CON (control or tap water). All units are in mg.

| **TRT** | **CYCLE** | **N** | **P** | **K** | **Ca** | **Mg** | **Cu** | **Zn** | **B** |
| --- | --- | --- | --- | --- | --- | --- | --- | --- | --- |
| N1 | 1 | 77.05 ± 0.56 | 39.17 ± 1.81 | 140.81 ± 2.87 | 41.59 ± 1.38 | 13.67 ± 0.33 | 0.047 ± 0.001 | 0.380 ± 0.009 | 0.086 ± 0.001 |
| N0.5R3 |  | 96.25 ± 1.34 | 40.68 ± 1.30 | 170.57 ± 1.87 | 50.62 ± 0.77 | 17.52 ± 0.45 | 0.056 ± 0.002 | 0.500 ± 0.004 | 0.116 ± 0.001 |
| R3 |  | 82.00 ± 2.68 | 33.58 ± 2.49 | 145.91 ± 3.14 | 47.93 ± 2.18 | 12.72 ± 0.79 | 0.046 ± 0.002 | 0.451 ± 0.024 | 0.123 ± 0.002 |
| CON |  | 41.81 ± 1.18 | 16.86 ± 0.97 | 75.48 ± 3.73 | 27.74 ± 0.89 | 6.87 ± 0.47 | 0.028 ± 0.002 | 0.266 ± 0.009 | 0.058 ± 0.002 |
| N1 | 2 | 74.51 ± 2.26 | 37.87 ± 0.83 | 137.44 ± 3.61 | 41.50 ± 1.91 | 13.49 ± 0.97 | 0.046 ± 0.001 | 0.377 ± 0.016 | 0.083 ± 0.004 |
| N0.5R3 |  | 92.43 ± 2.73 | 42.06 ± 0.77 | 162.41 ± 4.18 | 49.87 ± 1.86 | 17.10 ± 0.83 | 0.055 ± 0.002 | 0.492 ± 0.015 | 0.111 ± 0.002 |
| R3 |  | 79.24 ± 3.41 | 35.03 ± 2.89 | 139.32 ± 5.49 | 45.40 ± 2.33 | 12.58 ± 0.73 | 0.049 ± 0.002 | 0.439 ± 0.018 | 0.111 ± 0.003 |
| CON |  | 41.19 ± 2.58 | 17.68 ± 1.65 | 74.08 ± 3.42 | 27.39 ± 1.66 | 7.04 ± 0.55 | 0.030 ± 0.002 | 0.268 ± 0.017 | 0.057 ± 0.004 |
| N1 | 3 | 86.96 ± 2.83 | 44.74 ± 0.87 | 161.41 ± 3.44 | 49.30 ± 2.11 | 15.82 ± 0.76 | 0.053 ± 0.002 | 0.451 ± 0.011 | 0.098 ± 0.005 |
| N0.5R3 |  | 102.96 ± 2.57 | 50.46 ± 0.80 | 185.01 ± 5.31 | 57.75 ± 1.82 | 19.26 ± 0.74 | 0.064 ± 0.003 | 0.554 ± 0.009 | 0.127 ± 0.005 |
| R3 |  | 90.69 ± 3.45 | 44.26 ± 1.66 | 162.95 ± 5.76 | 51.81 ± 2.08 | 15.18 ± 0.49 | 0.058 ± 0.003 | 0.513 ± 0.017 | 0.126 ± 0.003 |
| CON |  | 54.55 ± 2.42 | 26.06 ± 1.54 | 90.56 ± 4.42 | 33.77 ± 1.98 | 8.65 ± 0.60 | 0.037 ± 0.002 | 0.355 ± 0.023 | 0.073 ± 0.005 |

Table A4. Means (±SE) of soil nutrient content and soil bacterial population (SBP) due to the four treatments: R3 (three-day fermented washed rice water), N1 (recommended rate of NPK 15:15:15 fertilizer at 450 kg ha^-1^), N0.5R3 (combined half the recommended rate of NPK and R3), and CON (control or tap water). All units are in mg kg^-1^. Unit for SBP is CFU × 10^6^ g^-1^ soil.

| **TRT** | **CYCLE** | **NH_4_-N** | **NO_3_-N** | **P** | **K** | **Ca** | **Mg** | **Cu** | **Zn** | **B** | **SBP** |
| --- | --- | --- | --- | --- | --- | --- | --- | --- | --- | --- | --- |
| N1 | 1 | 76.38 ± 0.65 | 22.48 ± 0.34 | 52.58 ± 3.47 | 119.80 ± 5.75 | 168.50 ± 0.96 | 50.15 ± 1.72 | 0.113 ± 0.005 | 0.338 ± 0.009 | 0.210 ± 0.004 | 1.615 ± 0.010 |
| N0.5R3 |  | 90.08 ± 2.35 | 33.25 ± 1.11 | 60.03 ± 1.06 | 185.50 ± 4.35 | 187.50 ± 3.57 | 73.05 ± 2.13 | 0.133 ± 0.006 | 0.415 ± 0.006 | 0.240 ± 0.004 | 1.825 ± 0.013 |
| R3 |  | 84.08 ± 3.32 | 29.13 ± 0.81 | 49.10 ± 1.46 | 165.80 ± 2.70 | 189.30 ± 4.17 | 69.73 ± 0.91 | 0.128 ± 0.005 | 0.388 ± 0.009 | 0.268 ± 0.005 | 1.965 ± 0.010 |
| CON |  | 57.58 ± 1.25 | 17.58 ± 0.66 | 35.18 ± 1.15 | 120.25 ± 2.56 | 138.75 ± 2.02 | 41.25 ± 3.12 | 0.113 ± 0.005 | 0.248 ± 0.018 | 0.180 ± 0.007 | 1.848 ± 0.008 |
| N1 | 2 | 78.10 ± 1.14 | 24.38 ± 0.41 | 54.08 ± 1.53 | 126.75 ± 4.19 | 170.25 ± 1.03 | 53.35 ± 2.35 | 0.138 ± 0.014 | 0.335 ± 0.010 | 0.228 ± 0.003 | 1.583 ± 0.032 |
| N0.5R3 |  | 91.95 ± 1.70 | 35.05 ± 0.65 | 61.05 ± 1.18 | 180.13 ± 3.96 | 187.00 ± 1.58 | 75.05 ± 2.04 | 0.180 ± 0.019 | 0.425 ± 0.005 | 0.250 ± 0.006 | 1.810 ± 0.012 |
| R3 |  | 84.80 ± 3.02 | 30.45 ± 1.01 | 53.15 ± 0.42 | 170.25 ± 1.80 | 192.80 ± 1.45 | 71.88 ± 1.22 | 0.153 ± 0.014 | 0.395 ± 0.013 | 0.275 ± 0.003 | 1.973 ± 0.005 |
| CON |  | 53.33 ± 0.93 | 17.08 ± 0.63 | 34.10 ± 0.85 | 119.50 ± 3.75 | 131.50 ± 2.50 | 38.95 ± 3.01 | 0.113 ± 0.005 | 0.240 ± 0.020 | 0.190 ± 0.009 | 1.823 ± 0.013 |
| N1 | 3 | 78.80 ± 0.81 | 25.10 ± 1.29 | 55.53 ± 1.70 | 133.75 ± 5.20 | 172.63 ± 4.76 | 56.20 ± 2.24 | 0.153 ± 0.010 | 0.378 ± 0.014 | 0.230 ± 0.004 | 1.565 ± 0.048 |
| N0.5R3 |  | 90.00 ± 1.57 | 36.33 ± 0.80 | 62.05 ± 2.72 | 176.80 ± 3.31 | 186.75 ± 3.38 | 75.18 ± 4.26 | 0.175 ± 0.006 | 0.410 ± 0.004 | 0.265 ± 0.005 | 1.775 ± 0.032 |
| R3 |  | 85.23 ± 2.36 | 33.28 ± 0.85 | 52.13 ± 1.04 | 167.25 ± 4.31 | 146.85 ± 43.55 | 69.48 ± 2.81 | 0.155 ± 0.003 | 0.380 ± 0.020 | 0.280 ± 0.007 | 2.108 ± 0.049 |
| CON |  | 53.60 ± 0.99 | 17.50 ± 0.34 | 33.90 ± 2.23 | 123.18 ± 2.20 | 131.00 ± 1.96 | 39.33 ± 2.88 | 0.128 ± 0.005 | 0.278 ± 0.018 | 0.198 ± 0.005 | 1.855 ± 0.017 |
